# Rapid, simplified microscale quantitative analysis of lignin H/G/S composition with GC–MS in glass ampules and glass capillaries

**DOI:** 10.1016/j.mex.2019.11.005

**Published:** 2019-11-07

**Authors:** Q Qi, Jiaqi Hu, Long Qu, Xiangning Jiang, Yang Gai, Sofia A. Valenzuela, Liwang Qi

**Affiliations:** a College of Biological Sciences and Biotechnology, Beijing Forestry University, Beijing, China; b College of Horticulture, China Agriculture University, Beijing, China; c University of Concepcion, Concepcion, Chile; d Research Institution of Forestry, Chinese Academy of Forestry, Beijing, China

**Keywords:** Rapid, simplified microscale quantitative analysis of lignin H/G/S composition with GC–MS in glass ampules and glass capillaries, GC–MS, Monolignol, Ampule, Capillary, Thioacidolysis

## Abstract

The characterization of monolignols H-, G- and S- units composition Vanholme et al. (2010) of lignin is important in agriculture, forestry, herb medicine, livestock, and health care research Vanholme et al. (2008) and Sticklen (2008). The conventional methods often require a great deal of samples and reagents and are time-consuming. Here, we present a newly developed method with fewer operations. The optimized method is suitable for detecting and characterizing lignin composition of cell wall in different plant species and has the advantages of:

• Avoiding the influence of plasticizer by plasticware and enhancing the accuracy of monolignols analysis.
• Lowering the required samples from grams to milligrams, and organic reagents from milliliters to microliters.
• Reducing the time required from a few days to 6 h.

## Graphical abstract

**Figure:**
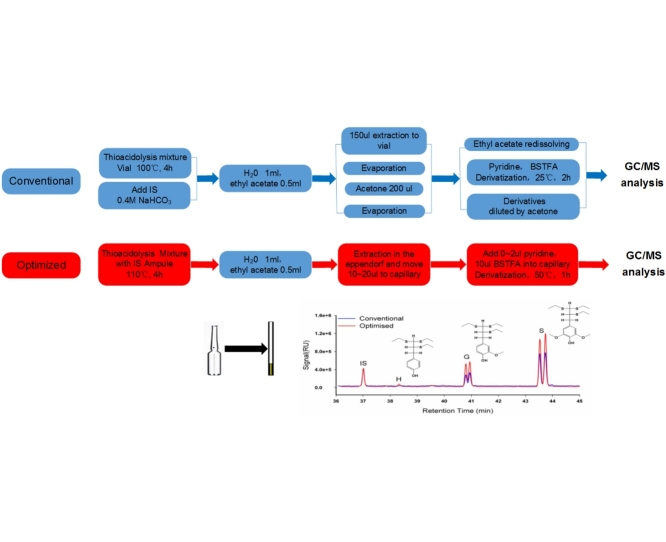

**Specification Table**

**Table:** 

Subject Area:	Agricultural and Biological Sciences
More specific subject area:	Forest Tree Physiology
Method name:	Rapid, simplified microscale quantitative analysis of lignin H/G/S composition with GC-MS in glass ampules and glass capillaries
Name and reference of original method:	Thioacidolysis of lignin: comparison with acidolysis *J Wood Chem. Technol.***5**, 1985, 277–292
Resource availability:	The data are available with this article

## Needs for monolignol analysis

Lignin is a complex compound mainly in secondary cell wall and associated with plant physiological functions, such as water transportation, physical support, and pathogen defense [1]. The resistance of lignin is not desirable for fodder, paper, herbal medicines, and biofuel production [2,3]. As a macromolecule, Lignin is polymerizate with p-coumaryl alcohol, coniferyl alcohol, and sinapyl alcohol [4]. Lignin removal is influenced by the proportion of monolignols and linkages. Therefore, lignin structure and linkage studies are important aspects of tree improvement.

Monomeric ratio varies remarkably among different tree species [5]. The 5-OH guaiacyl units (5-OH G units), ferulic, and p-coumaric acids are considered extra monolignols as they exist in the original structure of lignin at lower rates [6,7]. These monomers are linked through a variety of condensed (β-1, β-5, β-β, 5-5, 4-O-5) or uncondensed bonds (α/β-O-4). Among these bonds, β-O-4 is the most abundant and involved in 65–75 % of hardwood lignin basic structures [8]. Therefore, the efficient cleavage of an uncondensed β-O-4 bond is a prerequisite for the degradation of lignin structure into monomers and oligomers.

Numerous chemical degradation methods have been used over time to degrade lignin into low molecular weight products, such as acidolysis, permanganate oxidation, nitrobenzene oxidation, and the Derivatization Followed by Reductive Cleavage (DFRC) method [[9], [10], [11]]. Each chemical degradation method requires multiple analytical methods and instruments (*e.g.*, thin-layer chromatography (TLC), gas chromatography–mass spectroscopy (GC—MS), liquid chromatography–mass spectroscopy (LC—MS), gel permeation chromatography (GPC), and nuclear magnetic resonance (NMR)) [[12], [13], [14]]. Acidolysis is an early method for lignin degradation but has low yield and complicated chromatograms caused by side reactions [15]. During nitrobenzene oxidation, other *in situ* components such as phenolics are also oxidized, resulting in a misleading higher reaction yield [16]. Permanganate oxidation is effective only on the free-end group monolignol units and also has a specificity problem, as observed during nitrobenzene oxidation [9]. Although the DFRC method is reportedly highly specific and efficient, the DFRC degradation products contain β-O-4 structures and elemental bromine introduced after the reaction [17]. All the methods described have various issues and are time-consuming.

Thioacidolysis is a solvolysis method and the products are relatively stable and can be derivatized easily. Thus, thioacidolysis is widely used as a routine test for lignin composition research. Since first being introduced by Lapierre in 1985 [5] and generalized as a routine method by Rolando in 1992 [6], this method has been further developed and revised by many researchers [[18], [19], [20], [21]].

Although thioacidolysis is a powerful method, there are some drawbacks, which include (1) high minimum sample amounts required, (2) some harmful effects of ethanethiol, a major ingredient, on operators, (3) contaminating effects of organic reagents on the environment, (4) possible plasticizers and dyes on interfering substances by plastics, and (5) possible leaks from caps and therefore decreases of product yields.

A new method that is simple, rapid, precise, environmentally friendly and applicable at microscale is required. These are reflected in the optimized method of our study that refined the conventional thioacidolysis method to reduce sample requirement and reagent consumption, simplify operations, and produce high reaction yields and more reproducible results.

## Method details

Our optimized method provides significant improvements over the current thioacidolysis method in reaction volume, the amounts of toxic solvents used, the optimal ratio of derivatization reagents, the optimal conditions for reaction and derivatization, the replacements of plastic vessels and containers with glasses, and the simplified procedures of derivatization and sample processing.

### Container optimization

#### Replacements of plastic tubes and tools with glass containers (ampules, glass capillaries, and test tubes if needed) and tools (microsyringes and glass pipettes)

In general, a variety of organic reagents and solvents are required for microscale analysis [[5], [6], [7], [8]]. When in contact with plastic tubes and tips, impurities such as plasticizers and dyes may be introduced into samples, even though these tubes and tips are of high quality. The introduction of these contaminants could increase the complexity of chromatogram, decrease the accuracy of microanalysis, and give rise to side reactions. Such interferences and experimental errors from changing tips can be mostly avoided when glass appliances are used, which, at the same time, produces the more accurate and reproducible measurements of organic solvents, and reduces possible impacts on the environment and the operation time needed.

#### Our method is easy to operate

Procedures were simplified from about 14 steps to 5, and the processing time was shortened from days to hours. Sample throughput was enhanced, which provides the possibility of automated analysis. Multiple samples were measured in the same batch of workload.

#### High performance

Only glass containers and syringes were used in the procedure, and impurities such as plasticizers and dyes were not introduced. Reduced noise should lead to cleaner chromatograms such that minor/unique monomers are more likely to be detected. In addition, fused ampules and capillaries have better air-tightness, which should increase reaction efficiency. The thioacidolysis yield was twice as high as that of the control, without compromising reproducibility.

### Optimization of thioacidolysis reaction

#### Scaled down sample amount with high performance

**Fig. 1:**
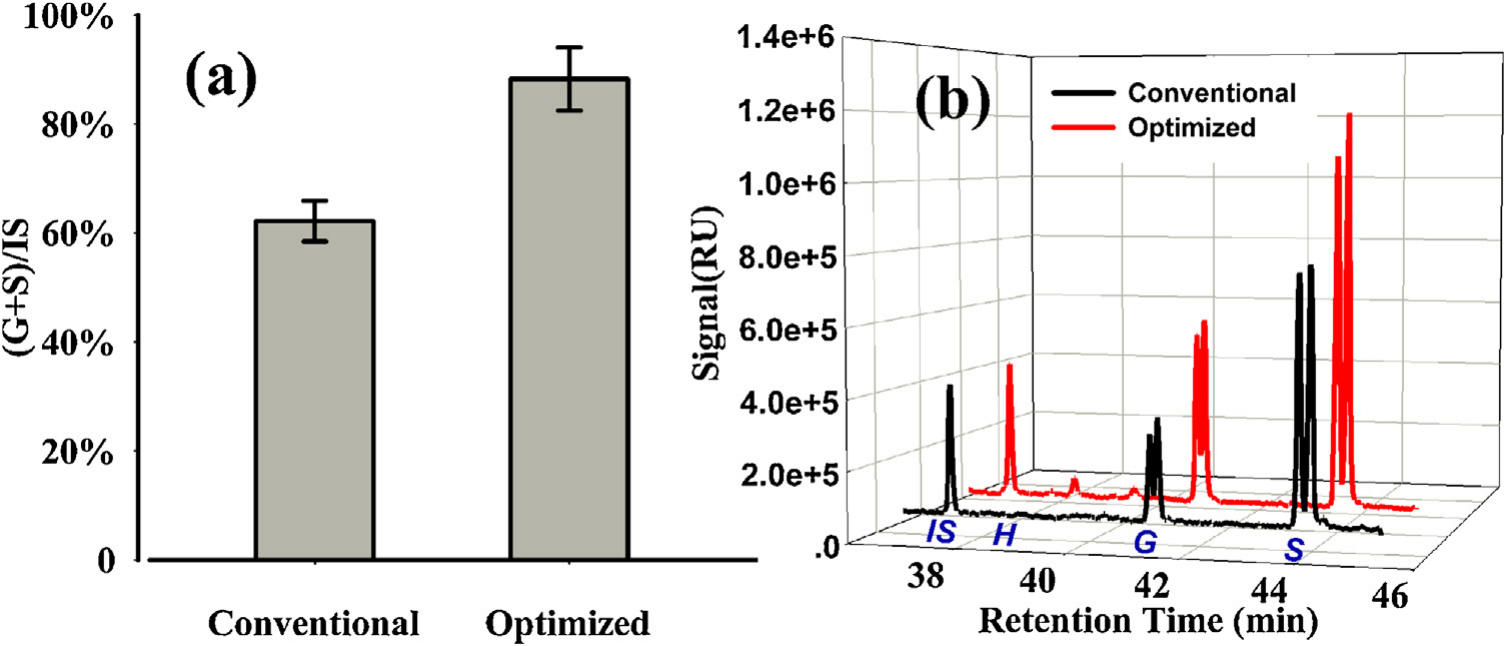
Lignin thioacidolysis yields with conventional and optimized method, (a) comparison of thioacidolysis yield between conventional and optimized method, and (b) gas chromatogram of angiosperm (*Populustomentosa*) thioacidolysis products, black line-conventional Method and redline-optimized Method. The total yield of optimized method is 131 % higher than that of conventional method.

The measurement of lignin monomers was not influenced by the reduced sample amount, while the total yield of lignin monomers was enhanced in terms of the relative signal (Fig. 1a) and chromatograms (Fig. 1b) detected. This suggests that the optimized method generates data more efficiently than the conventional method. Furthermore, the reduced sample requirement is important for biochemical analysis, especially for expensive samples or specimens that are difficult to obtain, such as tiny plant organs or rare mutants. The high sample requirement can restrict some experiments.

#### An internal standard added before heating

In this study, an internal standard C_24_ was added to the thioacidolysis mixture, which had two benefits. First, adding reaction mixture to samples helps stabilize the amount of internal standard in each ampule and reduce errors as opposed to adding the reagents individually after reaction. Second, this optimized step could effectively decrease the repetitive work of adding reagents.

**Fig. 2:**
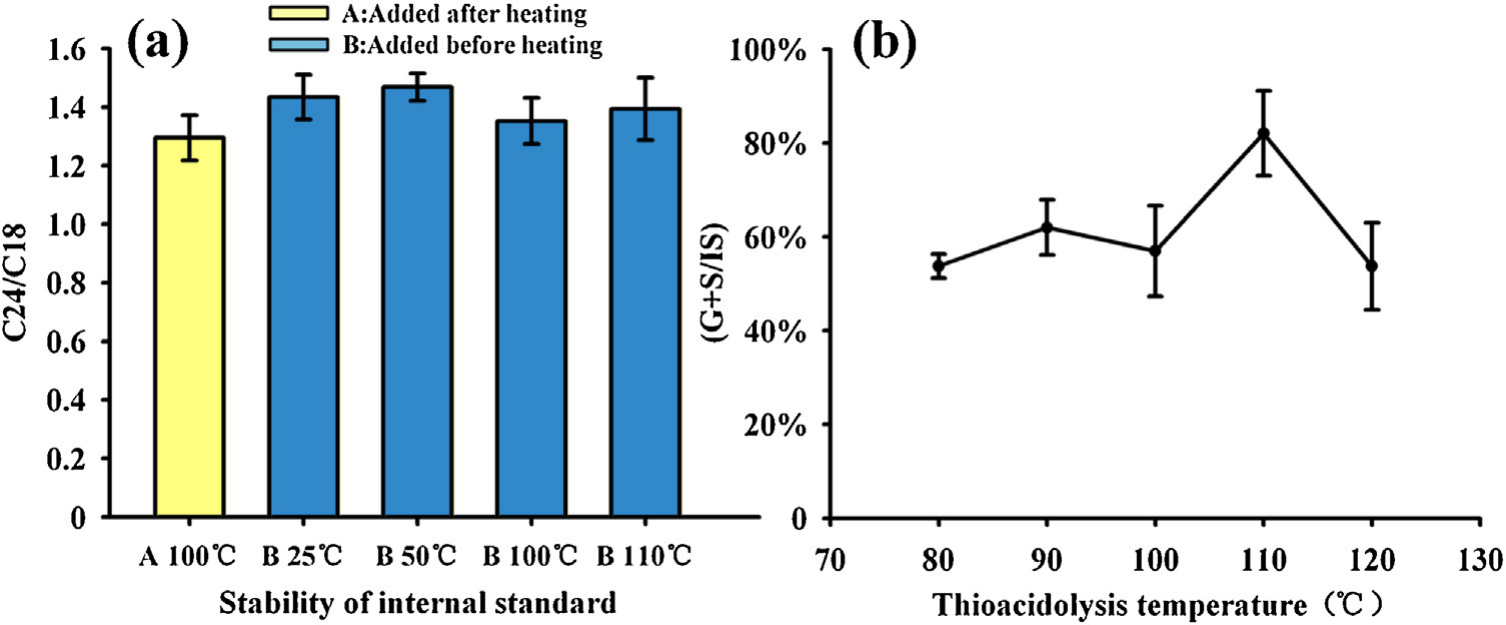
Optimization of temperature for internal standard stability and thioacidolysis reaction, (a) recovery of internal standard under different thioacidolysis temperatures and adding orders, and (b) optimized temperature of thioacidolysis reaction. Temperature and adding order didn’t affect recovery of internal standard and the highest monomer productivity was detected at 110°C.

Although adding an internal standard to the thioacidolysis mixture is beneficial and tetracosane is a stable alkane (with a boiling point of 391 °C), the solubility and stability of tetracosane at different temperatures in the thioacidolysis mixture is still needed. Thus, a confirmatory experiment is required. In this experiment, the recovery of tetracosane using the conventional method (adding tetracosane after heating) was defined as a control. The thioacidolysis mixture with the same amount of internal standard was heated to 25, 50, 100, and 110 °C. No significant difference was detected between experimental and control groups (four replicates) (Fig. 2a). Thus, adding an internal standard before heating is feasible.

#### Thioacidolysis reaction temperature optimization

Since the introduction of thioacidolysis by Lapierre in 1985 [5], the reaction temperature is commonly set at 100 °C according to acidolysis, which is generally considered satisfactory. Although thioacidolysis is commonly used in lignin research, the reactions at other temperatures have rarely been reporte. Here, we performed a systematical optimization experiment to determine the optimal reaction temperature for thioacidolysis with five reaction temperatures (80, 90, 100, 110, and 120 °C, with three replicates for each temperature). The results indicated a relative signal (G and S) of 80 % at 110 °C, which was better than that at other temperatures (Fig. 2b).

#### Water-saturated ethyl acetate as the extraction solvent

In the conventional methods, dichloromethane is commonly used as the extraction solvent. Because the dichloromethane phase is heavier than the water phase, the organic phase ends up with mixed solid impurities that introduce particles into the extraction. This operation leads to a more complex chromatogram and imprecise quantitative results. To address this problem, we used ethyl acetate as the extraction solvent that stayed in the upper layer with better separation from sediments. Additionally, water-saturated extraction solvent alleviates volume deviation from liquid inter-infiltration.

#### Optimization of extraction pH

**Fig. 3:**
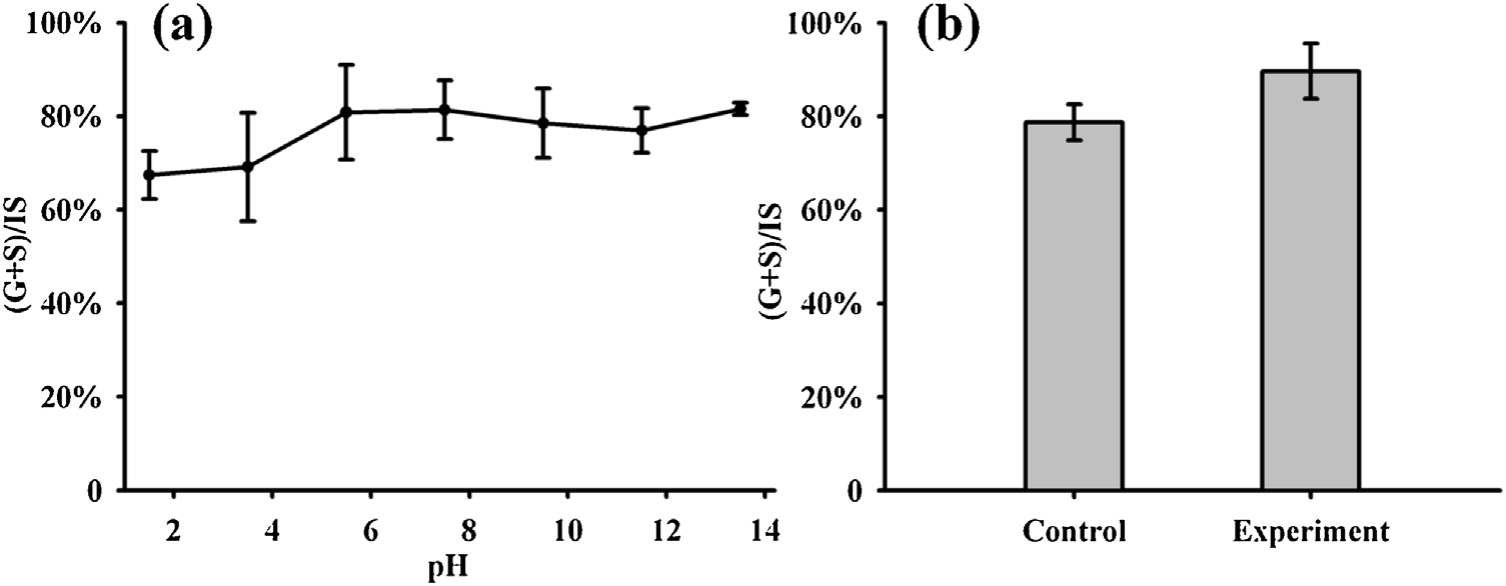
The effects of pH on extraction and derivatization, (a) optimized pH in extraction step, and (b) optimized pH in derivatization pretreatment. The pH of water phase didn’t influence recovery of products and derivatization directly was better than desiccation treatment.

The pH of the aqueous phase is usually adjusted to 3–4 before extraction in most thioacidolysis assays. However, further verification experiments have not been reported. A systematic experiment regarding the extraction pH was performed with our study. After thioacidolysis, the aqueous phase was adjusted to a pH of 1–2, 3–4, 5–6, 7–8, 9–10, 11–12, and 13–14 with HCl, NaHCO_3,_ and NaOH (three replicates). No significant difference was detected among treatment groups (Fig. 3a), indicating no need for pH adjustment. Skipping this step could enhance the efficiency and save time when performing these experiments.

#### Optimization of BSTFA concentration

**Fig. 4:**
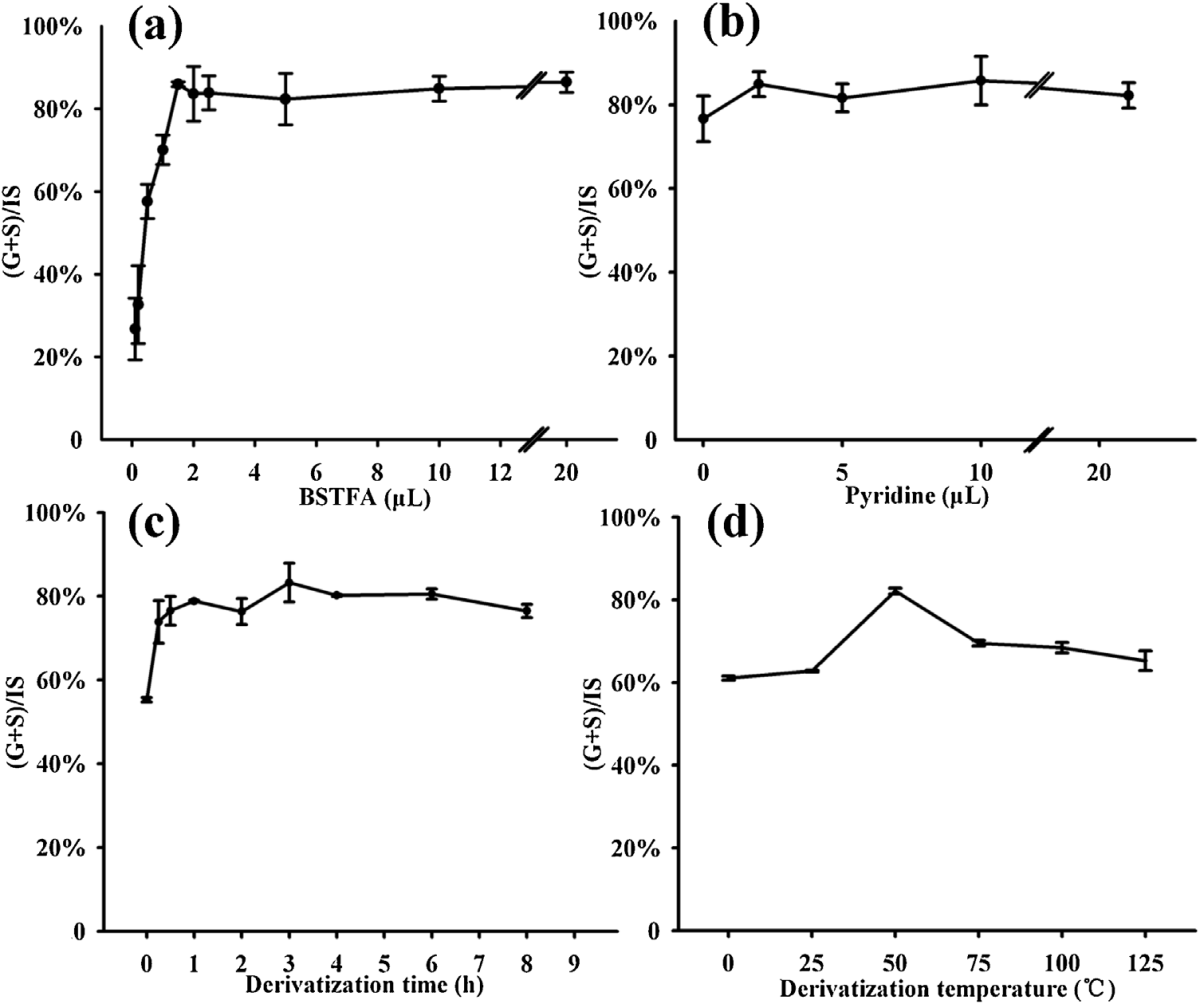
Optimized dosages of derivatization chemicals by derivatization time and temperature, (a) optimized BSTFA dosage, (b) optimized pyridine dosage, (c) optimized derivatization time, and (d) optimized derivatization temperature. It is shown that 10 μL of BSTFA was enough for full derivatization, the amount of pyridine didn’t influence derivatization yield, and optimized derivatization should be conducted at 50°C for 1 h.

BSTFA is the most common derivatization reagent used in lignin composition research [11]. As a basic principle, excessive amounts are generally added to ensure a complete reaction. As information on suitable amounts for optimization is limited, the reagent amount varies among researchers without a reasonable theoretical basis. The excessive amount of reagent reduces the relative concentration of products and results in reagent waste and environmental pollution. As such, a confirmatory experiment was performed to determine the minimum reagent required for complete derivatization and excessive addition. In the experiment (three replicates), 20 μL of extract (corresponding to 80 μg of wood meal) was transferred to capillary tubes, along with 0.1, 0.2, 0.5, 1.0, 1.5, 2, 2.5, 5, 10, and 20 μL of BSTFA. The final volume of each capillary tube was adjusted to 30 μL with ethyl acetate. Derivatization was conducted at 50 °C for 1 h. The results indicated the minimum amount of reagent at 1.5–2 μL of BSTFA and possible excessive addition at 10 μL and above (five times of the minimum amount) (Fig. 4a).

#### Optimization of pyridine amount

As for BSTFA, a confirmatory experiment was performed to optimize the amount of pyridine. A total of 0, 2, 5, 10, or 22 μL of pyridine was added to each capillary tube along with 10 μL of BSTFA (three replicates). The total volume was adjusted to 32 μL with anhydrous ethyl acetate. Again, no significant difference was detected among the treatment groups (Fig. 4b), indicating no influence of monomer yield by pyridine amount.

#### Optimization of derivatization time and temperature

Ten different derivatization time segments were tested, 0 min, 15 min, 30 min, 1 h, 2 h, 3 h, 4 h, 6 h, 8 h, and 24 h with three replicates. The yield was above 50 % immediately after well mixing, but stayed around 80 % with derivatization time from 15 min to 8 h (Fig. 4c). As the derivatization products decomposed heavily at 24 h (not shown in Fig. 4), the optimized derivatization time of 1-h was chosen.

A separate optimization experiment was performed to compare derivatization temperature at 0, 25, 50, 75, 100, and 125 °C (three replicates). The derivatization at 50 °C yielded the highest derivatives, which set 50 °C as the optimal temperature for derivatization (Fig. 4d).

#### Extraction procedure optimization

BSTFA (N, O-bis (trimethylsilyl) acetamide) is commonly used as a high-efficiency silylation reagent with many advantages, such as a high derivatization rate, short derivatization time, no strict temperature limit, and fewer derivatization byproducts. Because BSTFA is especially sensitive to moisture, the silylation with BSTFA needs to be conducted in an anhydrous environment. This requires the organic extraction of thioacidolysis products being dried with desiccants (*e.g.*, Na_2_SO_4_), which minimizes water influence, but results in sample loss.

We conducted an experiment to optimize the pretreatment of organic extraction before derivatization. In the control group, 20 μL of organic extraction was transferred to a glass capillary and evaporated in a dryer at 37 °C, dissolved with the same volume of anhydrous ethyl acetate assisted by ultrasonic oscillation for 24 h, and then derivatized with 10 μL of BSTFA. The experimental group was derivatized directly in the same way after transferring for 20 min (four replicates per group). The results showed a significantly higher yield with the experimental group (Fig. 3b), likely due to microscale extractions (*e.g.*, 20 μL), as residual water was small relative to BSTFA and losses during desiccation, and should not affect derivatization reaction.

#### References for further optimization in detecting H, G, and S ligninsfrom different plant materials

**Fig. 5:**
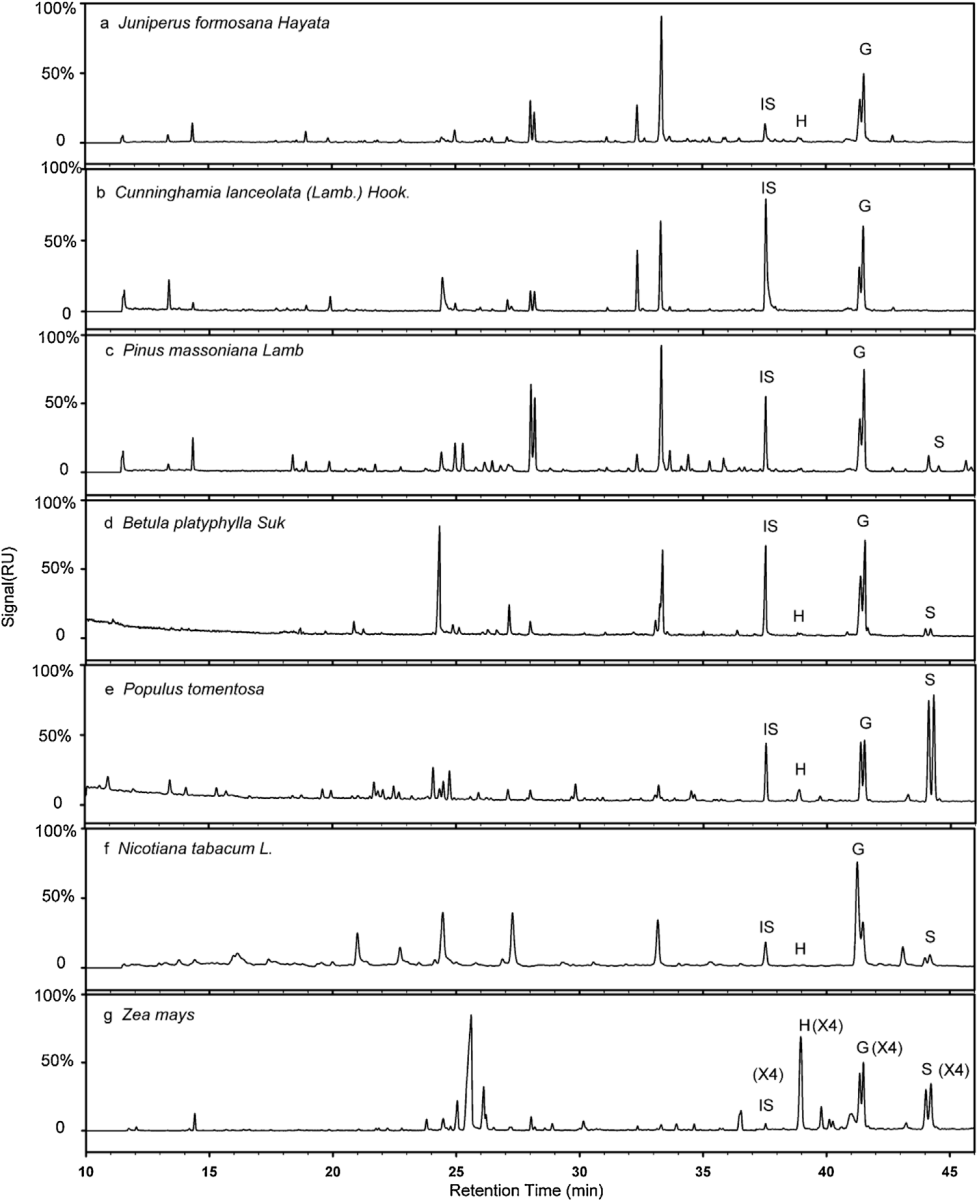
Total ion chromatograms of lignin H (p-Hydroxyphenyl monomer)/G (Guaiacyl monomer)/S (Syringly monomer) detected with the optimized method in seven tree species (a) *Juniperus formosana Hayata*, (b) *Cunninghamia lanceolata (Lamb.) Hook.*, (c) *Pinus massoniana Lamb*, (d) *Betula platyphylla Suk*, (e) *Populus tomentosa*, (f) *Nicotiana tabacum L*. (g) *Zea mays*.

Although the sample pretreatments, thioacidolysis reaction, and derivatization conditions may need to be adjusted in each laboratory, our tested approaches should be applicable for lignin composition analysis in general. In this report, seven types of plant materials (species) were examined with the optimized method (Fig. 5). *Populus tomentosa* had many types of lignin other than the three main types of H, G, and S (Fig. 5e). *Juniperus formosana Hayata* (Fig. 5a), *Cunninghamia lanceolata (Lamb.) Hook*. (Fig. 5b), and *Pinus massoniana Lamb* (Fig. 5c) contained monocotyledons but low S monomer, as the lignin in monocotyledons is mainly type G instead of S. *Betula platyphylla Suk* (Fig. 5d), *Populus tomentosa* (Fig. 5e), and *Nicotiana tabacum L*. (Fig. 5d) had high G and S, while *Populus tomentosa* (Fig. 5e) and *Betula platyphylla Suk* (Fig. 5e) were dicots and had different lignin constituents. *Populus tomentosa* and *Betula platyphylla Suk*. were dicots and had low H-type lignin; their S/G were 0.784 and 0.067 in respectively and H lignin was below 0.5 %. *Zea mays* had different constituents from the other six species; its S/G was 0.80 and S/H was 1.08 in corn, a monocot with a high concentration of H-type monomer (Fig. 5g). Thus, the optimized method described above is applicable to both dicots and monocots.
